# A Study on the Antimicrobial and Antibiofilm Peptide 1018-K6 as Potential Alternative to Antibiotics against Food-Pathogen *Salmonella enterica*

**DOI:** 10.3390/foods10061372

**Published:** 2021-06-14

**Authors:** Rossella Festa, Rosa Luisa Ambrosio, Alexandre Lamas, Lorena Gratino, Gianna Palmieri, Carlos Manuel Franco, Alberto Cepeda, Aniello Anastasio

**Affiliations:** 1 Department of Veterinary Medicine and Animal Production, University of Naples Federico II, 80137 Napoli, Italy; rossella.festa@unina.it (R.F.); rosaluisa.ambrosio@unina.it (R.L.A.); anastasi@unina.it (A.A.); 2 Laboratorio de Higiene Inspección y Control de Alimentos, Departamento de Química Analítica, Nutrición y Bromatología, Universidad de Santiago de Compostela, 27002 Lugo, Spain; alexandre.lamas@usc.es (A.L.); alberto.cepeda@usc.es (A.C.); 3 Institute of Biosciences and BioResources, National Research Council (IBBR-CNR), 80131 Napoli, Italy; lorena.gratino@ibbr.cnr.it; 4 Materias Srl, 80146 Naples, Italy

**Keywords:** antimicrobial peptide, *Salmonella*, food-borne pathogen, biofilm, 1018-k16, food preservatives

## Abstract

Antimicrobial resistance has become one of the major global public health concerns, and it is indispensable to search for alternatives to conventional antibiotics. Recently, antimicrobial peptides have received great attention because of their broad-spectrum antimicrobial activity at relatively low concentrations, even against pathogens such as *Salmonella enterica*, which is responsible for most food-borne illnesses. This work aimed at evaluating the antimicrobial and antibiofilm activity of the innate defense peptide, named 1018-K6, against *S. enterica*. A total of 42 strains, belonging to three different subspecies and 32 serotypes, were included in this study. The antibiotic resistance profile of all the strains and the cytotoxic effects of 1018-K6 on mammalian fibroblast cells were also investigated. Results revealed that MIC (minimum inhibitory concentrations) and MBC (minimum bactericidal concentrations) values were in the ranges of 8–64 μg/mL and 16–128 μg/mL, respectively, although most strains (97%) showed MICs between 16 and 32 μg/mL. Moreover, sub-inhibitory concentrations of 1018-K6 strongly reduced the biofilm formation in several *S. enterica* strains, whatever the initial inoculum size. Our results demonstrated that 1018-K6 is able to control and manage *S. enterica* growth with a large potential for applications in the fields of active packaging and water disinfectants.

## 1. Introduction

Salmonellosis is considered the second most reported gastrointestinal infection in humans in the European Union (EU) [1]. This is due to the ability of the *Salmonella* genus serotypes to spread among a variety of species (both humans and animals act as host organisms). In 2019, 87923 confirmed human salmonellosis cases were reported in the EU [1]. Indeed, the main sources of human infection in *Salmonella* outbreaks (17.9% of the total number of outbreaks) are contaminated foods, especially eggs and egg-derived products. Over the last few years, the annual number of strong-evidence food-borne outbreaks (FBOs) by eggs rose significantly, accounting for 37.0% [1].

Human infections caused by *Salmonella* are divided into typhoid forms, due to *S*. *Typhi* and *S. Paratyphi*, and non-typhoid forms, especially due to *S. Typhimurium* and *S*. Enteritidis, which are responsible for over 50% of total gastrointestinal infections [1]. Typically, non-typhoidal *Salmonella* causes self-limiting disease, although immunocompromised elderly or young individuals may manifest severe infections that need medical treatments over a long time with extended-spectrum antibiotics [2]. However, the widespread and imprudent use of antibiotics may cause the development of multidrug-resistant strains of *Salmonella* [3], which could be transferred through food-producing animals to humans [4].

In 2019, a high proportion of *Salmonella* isolates from human patients in the EU were revealed to be resistant to sulfonamides, tetracyclines and ampicillin [5]. The increasing resistance to the “critically important antimicrobials” (CIA) of highest priority, as defined by the World Health Organization, is alarming, especially in the case of ciprofloxacin, the third-generation cephalosporins (cefotaxime and ceftazidime) and/or the carbapenems whose inefficacy is mainly due to the extended-spectrum beta-lactamase (ESBL)/AmpC/carbapenemase-producing bacteria among *Salmonella* spp. [5]. As a result, *Salmonella* infections are becoming increasingly more difficult to treat, even with last-resort antibiotics. Therefore, in the era of novel technologies for innovative biomedical approaches, one of the main scientific challenges is the exploration of alternative solutions to the current inefficient treatments of infections with traditional antibiotics [6].

Moreover, recent studies revealed the social aspects of the life of bacteria, such as biofilm formation, which is mostly affected by environmental conditions. Biofilms play a crucial role in antimicrobial resistance phenomena, decreasing bacterial susceptibility to antibiotic action [7]. Indeed, the first case reported of food-borne bacterial biofilm infection was associated with *Salmonella* spp., whose biofilm-production has been largely studied and described [8]. The switch from a free-living state to the biofilm mode of growth allows bacterial cells to survive under adverse conditions, which may be recreated in chicken slaughterhouses and poultry farms [9]. Previous works described *S*. Enteritidis as one of the strongest biofilm-producer serotypes [10] and identified wood as the surface that allows the best bacterial adhesion [11]. As *Salmonella* biofilms show high resistance to disinfectants and antibiotics, it is necessary to develop innovative strategies to inhibit and/or prevent their formation. 

In this regard, antimicrobial peptides (AMPs) represent an exciting option taken progressively into consideration. AMPs have gained increasing attention as new potential antimicrobial drugs to replace or potentiate the action of conventional antibiotics in controlling infections caused by pathogens, due to their efficient activity and unique mechanism associated with specific chemical-physical properties [12,13,14]. AMPs are short amino acid sequences forming an ancient type of innate immunity found in all living organisms from bacteria to mammals, which work by providing a first line of defense against invading pathogens. Although there is a high number and wide sequence diversity of AMPs in nature, there are common structural features shared by the majority of these compounds [15]. These peptides are usually around 10–40 residues in length with net positive charges (from +2 to +9) and high extents of hydrophobic residues, ranging from 40 to 50%, arranged so that the folded peptide can adopt amphipathic structures [13,16]. As a result, AMPs display a strong tendency to establish non-specific interactions with negatively charged phospholipids, such as phosphatidylglycerol, which are typically abundant in the microbial cell membranes, leading to an increased permeability, leakage of cytoplasmic components and cell death [16,17]. In contrast to the majority of the available antibiotics that target specific biosynthetic pathways, AMPs efficiently kill microbial pathogens in most cases by accumulation on the membrane surfaces, pore formation and damage to the cell envelope integrity [17]. Therefore, AMPs can be effective against a wide spectrum of organisms, such as Gram-positive and Gram-negative bacteria, as well as fungi and viruses, and their non-specific mode of action significantly prevents the induction of resistance, as it is metabolically costly for most microbes to mutate or repair membrane components [17,18,19]. In addition, due to their role in the organisms as natural microbicides, AMPs are selectively cytotoxic to microorganisms, whilst they generally exhibit low or no toxic effects towards animal cells of the host organism. Indeed, the hemolytic activity and toxicity of these peptides were demonstrated to be linked to a range of physicochemical properties and to the membrane composition of the target cells. However, the cell selectivity issue needs to be seriously investigated for this class of antimicrobial compounds, as it is an important aspect that can limit their applications in vivo [20,21].

In a previous study, a cathelicidin-related antimicrobial peptide consisting of 12 residues and named 1018-K6 was in silico designed and characterized [22,23]. The peptide displayed high structural stability as well as powerful antimicrobial and antibiofilm activities at a low-micromolar range against Gram-positive and Gram-negative bacterial pathogens, including MRSA [23,24]. Moreover, preliminary toxicity assays showed that 1018-K6 did not affect the cell morphology of different human cell lines. In addition, 1018-K6 was revealed to adopt mixed α-helical/-β-sheet conformations when in contact with bacterial membrane mimics, and investigations on the antibacterial mechanisms strongly suggested that it belongs to the membrane-interacting peptide clan [23]. In light of these considerations, 1018-K6 represents a promising candidate for the development of a new generation of antibiotic molecules.

Herein, the bactericidal and antibiofilm activity of 1018-K6 against a large panel of serotypes of *Salmonella enterica* was investigated with the aim to explore the real potentials of this antimicrobial peptide further. The resistance profiles of all the strains included in this study were also determined. 

## 2. Materials and Methods

### 2.1. Strain Selection

The antimicrobial activity of 1018-K6 was evaluated against 42 different *Salmonella* strains, belonging to *S. enterica* subsp. *enterica*, *S. enterica* subsp. *salamae* and *S. enterica* subsp. *arizonae*. Of these, two reference strains were provided by the Spanish Type Culture Collection (CECT) and ten by the Spanish National Reference Laboratory (NRL) for salmonellosis in animals. Twenty-eight wild isolates were collected from fish (one), straw (one) and animal feces samples, of which many originated from different broiler farms. The ability of 1018-K6 to prevent biofilm formation was evaluated against a smaller panel of eleven of these species and serovars. Details about the strains investigated in this study are shown in Table S1.

Wild strains were isolated from bovine and chicken feces, fish and straw according to ISO 6579-1:2017 (ISO, 2017). Briefly, samples were mixed with buffered peptone water (Merck Millipore, Germany) at 1/10 *w*/*v* and incubated for 18 h at 37 °C. Then, a volume of 100 µL of the incubated sample was inoculated onto Rappaport-Vassiliadis semi-solid modified medium (Difco Laboratories, Franklin Lakes, NJ, USA) and incubated at 41 °C for 48 h for selective enrichment. Then, presumptive positive plates of Salmonella were picked with an inoculating loop, spread on xylose lysine deoxycholate agar (Oxoid Ltd., Hampshire, UK) and chromID Salmonella agar (SM-ID2, bioMérieux, Marcy-l’ÉEtoile, France) and incubated at 37 °C for 24 h. Presumptive Salmonella colonies from xylose lysine deoxycholate and SM-ID2 were cultured in Nutrient Agar (PanReac AppliChem, Spain) for 24 h at 37 °C. Isolates were confirmed by a latex agglutination test (Microgen, London, UK) and API 20E (bioMérieux) and serotyped using the Kauffman–White typing scheme for the detection of somatic (O) and flagellar (H) antigens with standard antisera (Bio-Rad Laboratories, Hercules, CA, USA).

### 2.2. Medium and Reagents

Muller-Hinton Broth (Panreac Applichem, Barcelona, Spain) and Nutrient Agar (Panreac Applichem, Barcelona, Spain) were used to revitalize and prepare the bacterial inocula. Species confirmation was performed on selective medium XLD (Oxoid, Hampshire, UK) and CHROMID SM2 (Biomérieux, Marcy-l’Étoile, France). Serial dilutions were performed with 0.85% saline solution (Sodium Chloride 99.85%, Acros Organics). Crystal Violet (Panreac Applichem, Barcelona, Spain) and Methanol (EMSURE ACS, ISO, Reag. Ph. Eur., Merck, Kenilworth, NJ, USA) were used for Biofilm Inhibitory Concentration (BIC) tests, respectively, to stain the bacterial cells and solubilize dye bound in each well of the microplate.

The derivative 12-mer peptide 1018-K6 was purchased from SynPeptide Co., LTD (Shanghai, China).

### 2.3. Antibacterial Activity Assay

Before each experiment, bacterial strains, frozen at −80 °C, were revitalized in Muller–Hinton Broth at 37 °C for 24 h and then grown on Nutrient Agar at 37 °C for 24 h. Working cultures were obtained by transferring isolated colonies to 0.85% saline solution and adjusting the turbidity to 0.5 McFarland, which is equivalent to approximately 1–2 × 10^8^ colony-forming units (CFU)/mL. Therefore, bacterial suspensions were diluted to obtain a concentration of 10^3^ CFU/mL in Muller-Hinton Broth, confirmed by spread-plating 100 µL on selective plate agar (aerobically incubated at 37 °C for 24–48 h).

The Minimum Inhibitory Concentration (MIC) is defined as the lowest concentration of the peptide at which no bacterial growth is detected. Minimum Bactericidal Concentration (MBC) is defined as the lowest concentration of peptide at which more than 99.9% of the bacterial cells are killed. To evaluate MIC and MBC, the method described by Colagiorgi and coworkers was adopted [24]. Each strain was exposed to different concentrations of peptide 1018-K6. The intermediate stock solution of the peptide was produced daily by sonicating the frozen stock and diluting it to a concentration of 256 µg/mL in Muller–Hinton Broth. Briefly, each well of the microplate was filled with 50 µL of bacterial suspension (1 × 10^3^ CFU/mL) and 50 µL of peptide solutions at decreasing concentrations (ranging from 256 µg /mL to 0.25 µg /mL). Then, the microplate was incubated at 37 °C for 20 h. Cell growth was evaluated by the unaided eyes [25]. After MIC determination, to evaluate the MBC values, 100 µL of bacterial suspension, in which no visible bacterial growth was observed, were seeded in Nutrient Agar plates, and the plates were then incubated for 24 h at 37 °C. The same analysis was performed using two-fold increased concentrations of peptide (with respect to MIC values). The antimicrobial test was replicated at least three times for all strains.

### 2.4. Inhibition of Biofilm Formation

Inhibition of biofilm formation was determined in 96-well flat-bottom polystyrene microtiter plates by using the method described by O’Toole [26]. Culture solution and peptide intermediate stock preparations were performed daily as described above. After revitalization, bacterial cultures were serially diluted to 5 × 10^4^ CFU/mL or 5 × 10^8^ CFU/mL, to prove peptide antibiofilm activity in two different bacterial suspensions. Considering the MIC values, lower doses of peptide were chosen (1/2 and 1/4 of the MIC value), bearing in mind that optimal bacterial growth conditions are required to allow biofilm production. Each well of the microplate was filled with 230 µL of 1018-K6 suspension in growth medium (Muller–Hinton Broth) and 20 µL of bacterial culture. Microplates were incubated for 24 h at 37 °C. At the end of incubation, the supernatant was poured off, and wells were washed three times with 300 µL of distilled water. Bacterial cells attached to the walls of the microplate were fixed by adding 250 µL of absolute methanol, left for 15 min, and then the methanol was discarded. Wells were air-dried, and the remaining bacteria were stained with 250 µL of 0.1% (*w*/*v*) crystal violet solution for 5 min. Wells were rinsed by placing the microplate under running water. Microplates were air-dried again, and the crystal violet contained within the biofilm was solubilized using 250 µL of 33% (*v*/*v*) glacial acetic acid per well. The Optical Density (OD) of each well was measured at 630 nm with a plate reader. The antibiofilm test was replicated at least three times for all strains for both inocula concentrations.

### 2.5. Antimicrobial Resistance Profile

The minimum inhibitory concentrations (MICs) of *Salmonella* strains included in this study were determined against 12 different antibiotics: ampicillin (from 1 to 128 µg/mL), chloramphenicol (from 2 to 64 µg/mL), ciprofloxacin (from 0.06 to 8 µg/mL), gentamicin (from 0.5 to 32 µg/mL), kanamycin (from 4 to 128 µg/mL), levofloxacin (from 0.5 to 32μg/mL), nalidixic acid (from 2 to 64 µg/mL), streptomycin (from 4 to 256 µg/mL), sulfamethoxazole (from 8 to1024 µg/mL), sulfisoxazole (from 8 to 1024 µg/mL), tetracycline (from 1 to 128 µg/mL) and trimethoprim (from 0.5 to 32 µg/mL). The standard antimicrobials were purchased from Sigma–Aldrich. MIC values were determined by using the broth microdilution method as described in the Clinical and Laboratory Standards Institute (CLSI) guidelines [26]. Briefly, for each test, antibiotic stocks of 4096 µg/mL were prepared in the diluent described in the CLSI guidelines and serially diluted in Mueller–Hinton broth (MH Broth, Panreac Applichem, Barcelona, Spain). All *Salmonella* strains were grown for 16–18 h at 37 °C in Nutrient agar (Panreac Applichem, Barcelona, Spain), and a single colony from incubated plates was picked and transferred to 10 mL of 0.85% saline solution at a final concentration of 0.5 McFarland. In the next step, the saline tube was serially diluted to a final *Salmonella* concentration of 10^6^ CFU/mL, and the 96-well microtiter plates were filled with 50 µL of each antibiotic dilution and 50 µL of the individual *Salmonella* strain. The microtiter plates were incubated for 24 h at 37 °C and after incubation, MIC values were calculated. For each antibiotic compound and *Salmonella* strain, MIC was determined as the lowest concentration in which no visual bacterial growth was observed. *Escherichia coli* (ATCC 25922) and *Enterococcus faecalis* (ATCC 29212) were used as control strains, and the susceptibility or resistance of each isolate was determined in agreement with the 2020 CLSI recommendations [25]. Moreover, all the *Salmonella* isolates, exhibiting resistance to at least three classes of antimicrobial agents tested, were considered multi-resistant.

### 2.6. Cytotoxicity Assays on Mammalian Cells

Neutral Red Uptake (NRU) assay was used to assay the potential 1018-K6 toxicity on mammalian fibroblasts BALB 3T3 clone A31 (ATCC CCL-163), at different peptide concentrations. Cell cultures were performed in Dulbecco’s Modified Eagle’s Medium (DMEM) supplied with 4 mM Glutamine and 10% Newborn Calf Serum. For the experimental assay (NRU), the seeding of BALB 3T3 clone A31 (ATCC CCL-163) cells was carried out in a 96-well microtiter plate (Thermo Fisher Scientific, Milan, Italy) incubated in the humidified environment and the presence of CO_2_ (5%) at 37 °C for 24 h. Incubations under these specific conditions allowed cell sedimentation and the establishment of a sub-confluent monolayer before supplying 1018-K6. Mammalian cells were treated with two doses of 1018-K6 (16 µg/mL and 80 µg/mL) for 24 h at 37 °C. Therefore, 150 µL of D-PBS with Ca2+/Mg2+ were used to rinse each well before exposing cells to 50 µg/mL of Neutral Red (NR) dye solution for 3 h at 37 °C. Then, after a second rinse (as described above), 150 µL of NR desorb solution (49% ddH2O, 50% ethanol, 1% acetic acid) were added to each well, and microplates were incubated in darkness under gentle agitation for 10 min. The OD of the NR extract was measured spectrophotometrically at 540 nm. 

Cell viability was expressed as the percentage of BALB 3T3 clone A31 cells that survived after treatment with 1018-K6. The parameter was compared to the control sample (mammalian cells grown in DMEM with 5% NCS, 4 mM Glutamine and 0.1% DMSO) which has a cell viability of 100%:

(OD treated cells − OD blank)/(OD Control Cells − OD blank) × 100

In order to analyze the obtained data, the ISO 10993-5:2009 was taken into account: the substance was identified as cytotoxic if the relative cell viability resulted to be <70% with respect to the control group, while the compound was classified as non-cytotoxic if the cell viability was ≥70% of the control sample.

### 2.7. Statistical Analysis

SPSS software version 26 (IBM Analytics, Armonk, NY, USA) was used to perform statistical analysis. Analysis of variance (generalized linear mixed model) was used to study the MBC values, the influence of the bacterial inoculum concentration on biofilm formation and the peptide effects at several doses on biofilm production for each serovar. An a posteriori contrast was performed using the Tukey test, considering a *p* value of <0.5 as statistically significant. 

## 3. Results and Discussion

### 3.1. Antimicrobial Activity of 1018-K6 on Planktonic Salmonella enterica Cells and Salmonella Resistance Profile

Recently, a 1018-derivative antimicrobial peptide named 1018-K6, (VRLIVKVRIWRR-NH2) was designed and characterized [22,23,24]. This 12-mer cationic peptide originates from the bovine host-defense peptide (HDP) bactenecins found in the neutrophil granules and belongs to the cathelicidin’s family. Structural and conformational studies performed on 1018-K6 clearly revealed that it showed a propensity to assume α-helix structures in membrane-mimetic models, such as micellar solutions of SDS [22,23,24]. In addition, this peptide retained its structural integrity in a wide range of pH and temperature conditions for prolonged incubation times, displaying also a significant bactericidal and antibiofilm activity against *Listeria monocytogenes* isolates from food products and contaminated environments [22,23,24]. 

**Figure 1:**
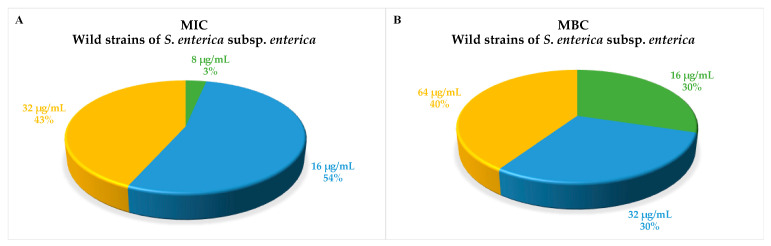
Distributions of Minimum Inhibitory Concentration (**A**) and Minimum Bactericidal Concentration (**B**) values among strains of *S*. *enterica* subsp. *enterica* are shown in the pie chart as a percentage of serovars related to a specific peptide dose.

In this work, the efficacy of 1018-K6 on the growth of a panel of wild and reference strains of different *Salmonella* serotypes was explored for potential applications in the food manufacturing industry. Taking into account the large amount of available experimental data on *Salmonella* spp. resistance to AMPs, the study was aimed at demonstrating the efficacy of 1018-K6 against this bacterial genus. Andersson et al. [27] accurately described *Salmonella* Typhimurium mechanisms of resistance (intrinsic and acquired) to cationic antimicrobial peptides. Therefore, the investigation on the peptide 1018-K6 was focused on testing its activity against a large number of strains to prove its efficiency as an antibiotic compound towards serovars, which are notoriously more resistant than others. For this reason, in order to obtain more representative results, more than 40 *Salmonella* strains across 32 different serotypes were involved in the experimental design. As reported in Table 1, a high variability, in terms of bacterial susceptibility to 1018-K6, was found among *Salmonella* subspecies and serovars. Nevertheless, each strain was characterized by very low minimum inhibitory concentration (MIC) values, ranging from 8 to 64 µg/mL. The highest concentration of the peptide needed to prevent visible bacterial growth was observed for *S*. *enterica* subspecies *salamae* 6,8: g, m, t (Table 1). Moreover, 1018-K6 was highly effective against 54% of the strains belonging to *S. enterica* subspecies, showing MIC values of 16 µg/mL (Figure 1A) towards serovars such as *S.* Enteritidis, *S.* Typhimurium, *S.* Infantis, *S.* Virchow and *S.* Hadar. 

A preliminary analysis of trends and correlations among the collected data from reference and wild strains (see Table S1 for bacterial source) evidenced an increase in antimicrobial performances of the peptide against the environmental *Salmonella* spp., especially towards *S. enterica* subspecies Enteritidis. These results are particularly relevant if we consider that some authors [27] have previously described *S*. Enteritidis and *S*. Typhimurium as the serovars more resistant than others to antimicrobial agents. Nevertheless, 1018-K6 appears to kill these pathogens efficiently, which are responsible for most of the human infection cases [5]. Moreover, the strains of *S.* Enteritidis and *S.* Typhimurium provided by the Spanish Type Culture Collection (CECT) (Table S1) showed higher MIC values than those determined for the same serovars isolated from chicken feces. This behavior is quite uncommon, as strains isolated from environments usually show a reduced susceptibility to antibiotics and disinfectants due to adaptation or resistance phenomena and the presence of mobile genetic elements carrying resistance genes or an altered permeability of the bacterial cell walls. However, further studies are needed to investigate the mechanisms underlying these aspects better. 

The concentration of the peptide able to kill the planktonic bacterial cells was also determined, obtaining the MBC values of 1018-K6 against the 42 *Salmonella* strains under investigation. The MBCs differed significantly between groups of strains ranging from 16 to 128 µg/mL when compared, with *S. arizonae* the most unaffected (Table 1). 

Once again, at least 50% of bacteria belonging to the subspecies *enterica* (Figure 1B) were sensitive to low doses (16–34 µg/mL) of the antimicrobial peptide, while also taking into account that a concentration of 16 µg/mL was sufficient to kill all *S.* Typhimurium monophasic cells. Interestingly, the antimicrobial results in Table 1 suggested a generally high correlation between MBC and MIC values of 1018-K6, with some tested bacteria even having identical values. This property is surprising and quite uncommon as the MBC of antimicrobial compounds is usually much higher than the corresponding MICs values, which indicates growth inhibition and not necessarily bacterial death and cannot distinguish between bactericidal and bacteriostatic effects. Indeed, the antibacterial agents are usually regarded as bactericidal if the MBC is no more than four times the MIC [28].

As previously described, increased resistance of bacteria to commonly used antibiotics has become one of the major challenges in the current medical practice. Therefore, to gain insight into the potential of 1018-K6 as antimicrobial, the antibiotic profile of the strains included in this study was investigated. Results revealed that three strains were multi-resistant, while S. Typhimurium monophasic exhibited the highest resistance profile, being resistant to four different antimicrobial groups, but showed a significant sensitivity to the action of 1018-K6 with low MIC and MBC values (16 µg/mL). Therefore, the peptide was remarkably effective even against multi-resistant strains at low concentrations. On the other hand, the effects observed against *S. enterica* subsp. *arizonae* 48:z4,z23 and *S. enterica* subsp. *arizonae* 48:z4,z23,z32 were less exciting, as evidenced by increased MIC values (128 µg/mL and >128 µg/mL) and no resistance of these strains to any antimicrobial investigated. However, serotypes of this subspecies are usually characterized by low virulence as they cause diseases only in highly immunosuppressed individuals with previous pathologies [29]. Hence, an inverse relationship may exist between the pathogenic potential of serotypes and the resistance to the peptide 1018-K6.

### 3.2. Evaluation of the Activity of 1018-K6 against Biofilm Formation of S. enterica

Due to the propensity of *Salmonella enterica* to attach to environmental and food matrixes [30], 1018-K6 was tested to counteract the biofilm-forming ability of a panel of pathogenic bacteria, as the biofilms represent an important virulence factor and the main source of environmental contamination [31]. The occurrence of clusters of bacteria bound to a surface and each other and embedded in a self-produced matrix is a common event in the food industry, and it has been noted that *S. enterica* under dry conditions can survive in a biofilm on stainless steel for over a year [32]. 

Biofilm inhibitory activity was evaluated against the *Salmonella* serotypes commonly recognized as a causative agent in food-borne outbreaks (*S. Enteritidis*, *S. Typhimurium*, *S. Infantis*, *S. Virchow* and *S. Hadar*) [1], and two reference strains. Among the tested strains, some microorganisms were chosen for their well-known ability in biofilm production (i.e., *S.* Typhimurium, *S.* Infantis) [33]. Therefore, based on optical spectroscopy, measurements of the amount of biofilm (i.e., OD_630nm_ readings following crystal violet staining) produced by each strain in the presence of sub-MIC concentrations of the peptide revealed that it led to significant reductions in biofilm growth. Interestingly, the amount of biofilm formed by *S. enterica* was not homogenous among serotypes investigated, and it did not depend on the inoculum concentration [34]. In fact, data on biofilm formation were compared to the initial planktonic cell concentration (Table S2) for each serotype, and an absence of a correlation was observed in at least 55% of strains (*S*. *Typhimurium*, *S*. *Typhimurium monophasic*, *S*. *Virchow*, *S*. *Dublin*, *S*. *Hadar* and *S*. *salamae*), whose variations in biofilm production were not significant, while differential responses were detected for *S*. Enteritidis CECT 4300, *S*. Enteritidis, *S*. Typhimurium CECT 4594, *S*. Infantis and *S*. Meleagridis (Table S2). Therefore, our study provides evidence for the first time that the initial cell concentration may not affect biofilm formation in different *Salmonella* spp.

**Figure 2:**
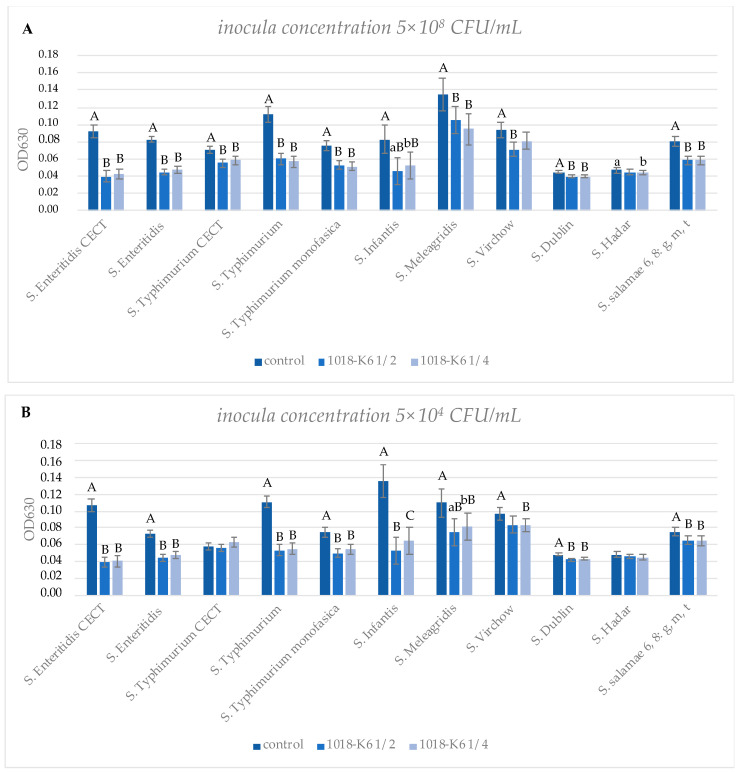
Antibiofilm activity of 1018-K6 on 11 strains of *S. enterica* from inocula of 10^8^ CFU/mL (**A**) and 10^4^ CFU/mL (**B**). Bacterial cells were grown under biofilm conditions in the absence (control) or presence of the peptide at a concentration of ½ and ¼ of the corresponding MIC values for each strain (1018-K6 ½ and 1018-K6 ¼, respectively). Statistical analysis was performed only within each strain, comparing the control with each treated bacterial sample. Average OD_630_ values are shown with error bars representing the standard error of four independent replicates. Different superscript uppercase letters indicate a significant difference within each strain at *p* < 0.01. Different superscript lowercase letters indicate a significant difference within each strain at *p* < 0.05.

Histograms (Figure 2) were used to illustrate the relationship between biofilm formation and peptide doses. The 1018-K6 differently decreased biofilm formation between species and serovars, and results obtained with the highest inoculum concentration (Figure 2A) indicated that it exhibited a significant inhibitory capacity versus all strains except for *S*. Hadar and *S*. Virchow. Moreover, among wild serovars, the peptide appeared to be more effective against *S*. Typhimurium and *S*. Enteritidis than the other isolates, significantly reducing biofilm mass by 49% and 44%, respectively.

Tests using inocula of 10^4^ CFU/mL confirmed the results reported above, despite the different initial concentration of planktonic cells (Figure 2B). Interestingly, in the case of *S*. *Typhimurium* CECT 4594, the lowest concentration of planktonic cells in the inoculum appeared to be better adapted to protection from the antimicrobial effects of 1018-K6, in contrast to that observed when a bacterial inoculum of 10^8^ CFU/mL was used (Figure 2) in the biofilm assays. Overall, more studies are necessary in order to clarify key factors affecting the antibiofilm activity of 1018-K6 and the complex mechanisms of biofilm formation among serovars better, taking into account that many features can interfere in the process such as the origin of the isolates [35].

Furthermore, bacterial biofilm reduction was found to be concentration-independent for many strains investigated (Figure 2), suggesting that a very low amount of 1018-K6 was necessary to inhibit the biofilm formation, and this effect appeared not to be dose-dependent. Therefore, these analyses validated the peptide efficacy on biofilm development, defining a great potentiality that might be further examined. In the last few years, several research studies explored new strategies to identify alternative antibiofilm substances able to replace the traditional compounds that often require high doses to obtain biofilm eradication/inhibition [36].

All these findings appeared in agreement with the previous studies [23,24] that designated 1018-K6 as an optimal antimicrobial candidate for its great activity against bacteria of the genus *Listeria* (i.e., *L. monocytogenes*) and *Staphylococcus* (i.e., *S. aureus* MRSA and *S. aureus* MSSA). Results reported in these works strongly supported the hypothesis that 1018-K6 is able to act via a cell-membrane-destabilization mode of action, which does not involve the interaction and inhibition of intracellular messengers of biofilm. However, secondary mechanisms of action of peptide could not be excluded, and further investigations are necessary to clarify these aspects better. 

### 3.3. Evaluation of Cytotoxic Effects of 1018-K6 on Mammalian Fibroblast Cells

The cytotoxic potential activity of 1018-K6 on mammalian cells was mostly analyzed in vitro by the Neutral Red Uptake (NRU) assay using the mammalian BALB 3T3 clone A31 fibroblast cell line. The NRU test is a common method to quantify the cytotoxicity of different chemical compounds in cell cultures. The principle of the assay is based on the uptake of the neutral red dye which accumulates in the lysosomes of uninjured cells. The NRU in vitro analysis is usually used to investigate initial doses for in vivo acute oral systemic toxicity tests. In addition, the BALB/3T3 mouse fibroblast cells were selected in our cytotoxicity study since the BALB/3T3 system was revealed to be very sensitive to a large panel of potential carcinogens or pro-carcinogens [37,38]. 

Based on these analyses, cell viability results, following the treatment with two different concentrations of 1018-K6 (16 and 80 µg/mL), were 99.9% and 97%, respectively, as determined by using the equation (1) reported in the Material and Methods section. Therefore, for Balb/3T3 cells, increasing doses of the antimicrobial peptide led to minimal inhibition of cell growth. These findings suggested that 1018-K6 did not exhibit any cytotoxic effect against the mammalian cells at the tested concentrations, even at of 80 µg/mL, which was sufficiently high to kill almost all the investigated pathogenic bacteria, except for *S*. *arizonae* 48:z4,z23 and *S*. *arizonae* 48:z4,z23,z32, which showed a MBC value of 128 µg/mL. It is noteworthy that 1018-K6 showed high selectivity towards bacteria over mammalian cells, possibly due to the different complex membrane lipids in these cells. In fact, the substantial variation in the composition of eukaryotic membranes in comparison to prokaryotic cell envelopes could explain the great selectivity of 1018-K6 for microbial cells. Indeed, it has been widely reported that the cationic antimicrobial peptides can preferentially bind to the negatively charged phospholipid bilayers of bacterial cells compared to the more neutrally charged eukaryotic cells [16,39]. These results further evidence the potential advantages that can be afforded by using 1018-K6 formulations in biotechnological and clinical applications.

## 4. Conclusions

The number of antibiotic-resistant pathogens is growing, and the capacity of the currently available antimicrobial strategies to manage bacterial infections is weakening. AMP can represent an alternative to conventional antibiotics to control and combat bacterial infections. In this study, we demonstrated that the peptide 1018-K6 displayed a potent efficacy against wild and reference strains belonging to *Salmonella enterica* subsp. *enterica*, including pathogens found in fish and poultry processing plants. Specifically, results revealed that 1018-K6 was effective at low concentrations, both in planktonic cells and biofilms of all strains investigated, including the multi-drug resistance bacteria that have engaged the scientific community in research for finding innovative and improved solutions. Therefore, due to the wide susceptibility profile, 1018-K6 could be proposed as a promising candidate for developing bio-sanitizing formulations or active packaging, making it applicable at several points in the food chain. Indeed, the remarkable stability and the broad-spectrum activity of this peptide was previously demonstrated, even in strong alkaline and acid environments [23], thus suggesting its use in sanitizer formulations equipped with acid or alkaline chemical ingredients to improve food sanitation and safety. Moreover, 1018-K6 has been efficiently bound to PET polymer materials to obtain antimicrobial-active packaging, showing potent antibacterial and anti-adhesion properties and the ability to control the alteration processes in food matrices [40,41]. Hence, the peptide could help in preventing the bacterial contamination during the transformation and production phases, being able to act downstream and upstream of the food chain.

However, further investigations are needed in order to elucidate the mode of action of this peptide against bacterial pathogens, such as the Gram-negative *Salmonella* spp. Indeed, the membrane permeability and destabilization have been previously published as the mechanism to describe the activity of the cationic AMPs, such as the peptide 1018-K6 investigated in our study. Specifically, the extracellular membrane of Gram-negative bacteria comprises negatively charged LPS. The cationic AMPs can replace the divalent cations linked to this unique lipopolysaccharide, cause a breakage or a pore on the cell outer membranes and finally go through extracellular membranes, inducing their disintegration and cell death [13,42]. Therefore, as we previously demonstrated [23,24] that the peptide 1018-K6 displays a membrane destabilization and pore-forming activity against two different Gram-positive microorganisms, it may be reasonable to suggest a similar model of mechanism for the peptide against the Gram-negative bacteria belonging to the *Salmonella* genus. 

## Figures and Tables

**Table 1:** MIC and MBC values of 1018-K6 against 42 *Salmonella* subspecies/serovars and analysis of their antibiotic resistance profiles.

	MIC	MBC	Antimicrobial Resistance Profile
**Subspecies or serovar**	**µg/mL**	**µg/mL**	**Resistant to**
*S*. Stanleyville	8	32	-
*S*. Agama	16	32	-
*S*. Anatum	16	16	-
*S*. Bredeney	16	64	-
*S*. Cerro	16	16	-
*S*. Dublin	16	16	-
*S*. Eboko	16	16	-
*S*. Enteritidis	16	64	Amp
*S*. Hadar	16	32	Tet, sulf
*S*. Infantis	16	16	-
*S*. Jerusalem	16	16	Sulf
*S*. Mbandaka	16	64	-
*S*. Mikawasima	16	16	Amp
*S*. Montevideo	16	64	Sulf
*S*. Newport	16	16	Sulf
*S*. Richmond	16	16	-
*S*. Seftenberg	16	16	Strep
*S*. Typhimurium monophasic	16	16	Tet, Strep, sulf, Amp
*S*. Typhimurium	16	64	
*S*. Typhimurium 1	16	64	Tet, strep, amp
*S*. Typhimurium 2	16	64	Amp
*S*. Typhimurium 3	16	64	Amp, Strep
*S*. Typhimurium 4	16	64	Strep
*S*. Virchow	16	32	Na
*S*. Isangi	32	64	Sulf
*S*. Meleagridis	32	32	Strep, sulf, Amp
*S*. Barro	32	32	-
*S*. Dabou	32	32	-
*S*. Drac	32	32	-
*S*. Enterica 4:b	32	32	Strep
*S*. Enteritidis CECT 4300	32	64	-
*S*. Ndolo	32	32	-
*S*. Poona	32	32	-
*S*. Thompson	32	64	Amp
*S*. Typhimurium CECT 4594	32	64	-
*S*. Typhimurium 5	32	64	Strep
*S*. Typhimurium 6	32	64	-
*S*. Typhimurium 7	32	64	-
*S*. Typhimurium 8	32	64	-
*S. arizonae* 48:z4,z23	32	>128	-
*S. arizonae* 48:z4,z23,z32	32	128	-
*S. salamae* 4, 12: b-	32	64	Sulf
*S. salamae* 4,5,12:b	32	64	Sulf
*S. salamae 6,8: g, m, t*	64	64	-

Amp: ampicillin; Na Nalidixic acid; Strep: streptomycin; Sul: sulfamethoxazole; tet: tetracycline. All the experiments were performed in triplicate. The MICs and MBCs values against *Salmonella* isolates were statistically analyzed using an ANOVA test. The modes were equivalent to the medians.

## Data Availability

The data presented in this study are available on request from the corresponding authors.

## Supplementary Materials

The following are available online at https://www.mdpi.com/article/10.3390/foods10061372/s1, Table S1: ΔOD630nm values (averaged of four independent replicates) of microbial biofilms produced by Salmonella *enterica* strains. Values are means ± standard error (ES). Table S2: Source of *Salmonella enterica* strains and scheduled tests.
