# Supplementary material for: A Study on the Antimicrobial and Antibiofilm Peptide 1018-K6 as Potential Alternative to Antibiotics against Food-Pathogen Salmonella enterica
Source: Foods. 2021 Jun 14;10(6):1372. doi: 10.3390/foods10061372 (PMC8232012; doi:10.3390/foods10061372)
Supplement: Supplementary file 1 [file foods-10-01372-s001.zip › foods-1220803-supplementary.pdf]

foods

MDPI

Table S1. Source of *Salmonella enterica* strains and scheduled tests.

**Salmonella** ***subspecies and serovar***	**Source**	**MIC**	**MBC**	**BIC**
S. Enteritidis CECT 4300	CECT	✓	✓	✓
S. Typhimurium CECT 4594	CECT	✓	✓	✓
S. Agama	Spanish NRL for Salmonellosis in animals	✓	✓	
S. Dublin	Spanish NRL for Salmonellosis in animals	✓	✓	✓
S. Eboko	Spanish NRL for Salmonellosis in animals	✓	✓	
S. Hadar	Spanish NRL for Salmonellosis in animals	✓	✓	✓
S. Infantis	Spanish NRL for Salmonellosis in animals	✓	✓	✓
S. Jerusalem	Spanish NRL for Salmonellosis in animals	✓	✓	
S. Meleagridis	Spanish NRL for Salmonellosis in animals	✓	✓	✓
S. Poona	Spanish NRL for Salmonellosis in animals	✓	✓	
S. Richmond	Spanish NRL for Salmonellosis in animals	✓	✓	
S. Virchow	Spanish NRL for Salmonellosis in animals	✓	✓	✓
S. Anatum	Bovine faeces	✓	✓	
S. Bardo	Chicken faeces	✓	✓	
S. Bredeney	Chicken faeces	✓	✓	
S. Dabou	Chicken faeces	✓	✓	
S. Drac	Chicken faeces	✓	✓	
S. Enteritidis	Chicken faeces	✓	✓	✓
S. Isangi	Chicken faeces	✓	✓	
S. Mbandaka	Chicken faeces	✓	✓	
S. Mikawasima	Chicken faeces	✓	✓	
S. Montevideo	Chicken faeces	✓	✓	
S. Ndolo	Chicken faeces	✓	✓	
S. Newport	Chicken faeces	✓	✓	
S. Seftenberg	Chicken faeces	✓	✓	
S. Stanleyville	Chicken faeces	✓	✓	
S. Thompson	Chicken faeces	✓	✓	
S. Typhimurium	Chicken faeces	✓	✓	✓
S. Typhimurium 1	Chicken faeces	✓	✓	
S. Typhimurium 2	Chicken faeces	✓	✓	
S. Typhimurium 3	Chicken faeces	✓	✓	
S. Typhimurium 4	Chicken faeces	✓	✓	
S. Typhimurium 5	Chicken faeces	✓	✓	
S. Typhimurium 6	Chicken faeces	✓	✓	
S. Typhimurium 7	Chicken faeces	✓	✓	
S. Typhimurium 8	Chicken faeces	✓	✓	
S. arizonae, serotype 48: z4, z23	Chicken faeces	✓	✓	
S. arizonae, serotype 48: z4, z23, z32	Chicken faeces	✓	✓	
S. salamae, serotype 4, 12:b-	Chicken faeces	✓	✓	
S. salamae, serotype 6, 8:g, m, t	Chicken faeces	✓	✓	✓
S. Cerro	Fish	✓	✓	
S. Typhimurium monofasica	Straw (cattle feed)	✓	✓	✓

Table S2. ΔOD_{630nm} values (averaged of four independent replicates) of microbial biofilms produced by *Salmonella enterica* strains. Values are means ± standard error (ES).

**Salmonella *serotypes***					**Biofilm formation**		
					Inoculum 10^8 CFU/mL	Inoculum 10^4 CFU/mL	
Genus	Species	Subsp.	Serovar	Source	Average OD_{630} ± ES	Average OD_{630} ± ES	*p*
Salmonella	*enterica*	*enterica*	Enteritidis 4300	CECT	0.092 ± 0.007	0.107 ± 0.007	0.008
Salmonella	*enterica*	*enterica*	Enteritidis	wild	0.083 ± 0.004	0.073 ± 0.004	0.002
Salmonella	*enterica*	*enterica*	Typhimurium 4594	CECT	0.071 ± 0.004	0.058 ± 0.004	7.47E-08
Salmonella	*enterica*	*enterica*	Typhimurium	wild	0.112 ± 0.009	0.111 ± 0.007	0.829
Salmonella	*enterica*	*enterica*	T. monofasica	wild	0.075 ± 0.006	0.074 ± 0.006	0.722
Salmonella	*enterica*	*enterica*	Infantis	NRL	0.083 ± 0.017	0.136 ± 0.02	0.001
Salmonella	*enterica*	*enterica*	Meleagridis	NRL	0.135 ± 0.019	0.11 ± 0.017	0.028
Salmonella	*enterica*	*enterica*	Virchow	NRL	0.094 ± 0.009	0.097 ± 0.008	0.574
Salmonella	*enterica*	*enterica*	Dublin	NRL	0.045 ± 0.002	0.048 ± 0.002	0.115
Salmonella	*enterica*	*enterica*	Hadar	NRL	0.047 ± 0.003	0.048 ± 0.003	0.584
Salmonella	*enterica*	*salamae*	6, 8: g, m, t	wild	0.081 ± 0.006	0.075 ± 0.005	0.169

Statistical analysis was performed only within each strain, comparing controls of different inoculum concentration.
